# Contingent Electric Skin Shock: An Empirical or Ideological Issue?

**DOI:** 10.1007/s40614-023-00380-3

**Published:** 2023-06-26

**Authors:** Nathan Blenkush, Dawn A. O’Neill, John O’Neill

**Affiliations:** The Judge Rotenberg Educational Center, 250 Turnpike Street, Canton, MA 02021 USA

**Keywords:** Severe behavior, Self-injury, Aggression, Shock

## Abstract

Intractable self-injury, aggressive, and other destructive behaviors are real human conditions. Contingent electric skin shock (CESS) is a technology, based on behavior-analytic principles, used to ameliorate such behaviors. However, CESS has always been extraordinarily controversial. The Association for Behavior Analysis (ABAI), commissioned an independent Task Force to examine the issue. After a comprehensive review, the Task Force suggested the treatment should be available for use in select cases through a largely accurate report. Yet, ABAI adopted a position indicating CESS is never appropriate. On the issue of CESS, we are extremely concerned behavior analysis departed from the fundamental epistemology of positivism and is misleading nascent behavior analysts and consumers of behavioral technology. Destructive behaviors are extremely difficult to treat. In our commentary, we outline clarifications regarding aspects of the Task Force Report, proliferation of falsehoods by leaders in our field, and limitations to the standard of care in behavior analysis. We recommend using science to answer important questions instead of propagating false information at the expense of current and future clients with treatment refractory behaviors.

Treatment refractory destructive behaviors are real, horrendous, and consume the lives of afflicted clients and their families. Consider “Ms. A” who emitted 123,784 destructive behaviors, endured 5,354 violent restraints, caused 343 staff injuries and was unsuccessfully treated with 20 psychopharmacological interventions, protective equipment, and 329 behavioral treatment variations across decades of treatment (see Blenkush & Cunningham, 2023). The behaviors resulted in immeasurable anguish and despair for the client and her family. In 2019, contingent electric skin shock (CESS) immediately eradicated her lifelong clinical problem behaviors and ended treatments with minimal benefits and iatrogenic effects, a treatment miracle. The Association for Behavior Analysis International (ABAI) Position Statement on the use of CESS (hereinafter “the Position Statement”) (Association for Behavior Analysis International [ABAI], 2022b), purportedly informed by a CESS Task Force Report (hereinafter “the Report”) (ABAI, 2022a), opposes this treatment and outcome. On one hand, the Position Statement affirms a client’s right to effective treatment and dignity and on the other would strip away individual rights and dignity through the condemnation of a treatment known to be efficacious and life changing. The bottom line finding of the Task Force was: “At this time, however, our assessment of the evidence – in the literature and in the clinical records we reviewed at the JRC [described in our Report] – does not support a wholesale ban of the procedure.” (ABAI, 2022a, p.6).

We found the Report to be objective and largely accurate. We applaud the work of the Task Force members. In this commentary, we offer (1) clarifications regarding aspects of the Report; (2) concerns about the proliferation of demonstrably false information; and (3) a summary of limitations to the standard of care and alternative approaches to treatment-refractory problem behavior.

## Clarifications

The Report states “Assent is not obtained from clients for the use of CESS” (p. 10). This statement is inaccurate. Assent is part of a comprehensive substituted judgement legal process designed to protect an incapacitated person during extraordinary treatments (e.g. intrusive behavior modification, antipsychotic medication, removal of maintenance nutrition) (Sherman, 1991). On an individual basis, before CESS is authorized, a Massachusetts Probate Court Judge considered (a) the client’s expressed preferences regarding treatment; (b) religious convictions; (c) the impact on the client’s family; (d) adverse side effects of the proposed treatment; (e) prognosis without treatment; (f) prognosis with the proposed treatment; and (g) other factors germane to the client’s case. An independent attorney was appointed to represent the needs of the client. The attorney’s role is to “…diligently and zealously advocate on behalf of his or her client…” (CPCS, 2022). Every substituted judgment treatment plan contains a statement of the clients expressed preference for treatment. Clients also expressed their preference to their assigned attorney. Many clients attended their hearing and spoke directly to the presiding Judge. Non-incapacitated clients provided written informed consent for CESS treatment. JRC has utilized this legal process since 1985, 35 years before assent was part of the Behavior Analyst Certification Board ethics code. Across the nation, similar protections are often not available for commonly utilized restrictive procedures (e.g. psychopharmacology, restraint, protective equipment).

The Report accurately described the range of temporal relations between a given response and CESS application across specific clinical contexts. There is a rationale behind the selected intervals which was not fully articulated in the report. When using CESS, clinicians must balance procedures that minimize treatment errors and those that maximize treatment efficacy. When CESS is introduced, a pre-verification procedure maximizes treatment efficacy until the behaviors reach low levels. Subsequently, a standard verification process prevents errors in CESS administration at the expense of temporal contiguity. If the standard verification has a negative impact on the client, the pre-verification procedure is re-introduced. Although 2 min is the upper limit by policy, in practice, the interval is much shorter, bridged by stimuli, and evaluated for efficacy on an individual basis.

The Report was an otherwise generally accurate reflection of the issue at hand. We turn now to a discussion of unsubstantiated claims and the propagation of false information by professionals in applied behavior analysis referenced in the Report.

## Proliferation of Falsehoods

Punishment procedures have been controversial for decades and research interest has dwindled, leaving empirical and theoretical gaps in the literature. Critics of punishment often cite Sidman (1989) as the seminal text on the reasons to avoid its use. However, many of those assertions and arguments against punishment have been called into question as lacking strong empirical support (Fontes & Shahan, 2020). Furthermore, the benchmark textbook in applied behavior analysis (Cooper et al., 2020), referenced in the Report, stated:

Even the most serious life-threatening behaviors can be treated effectively and humanely without resorting to the use of noxious or painful unconditioned punishers (e.g., water mist, aromatic ammonia, electric shock). Research has shown that the judicious use of [alternative procedures] can reduce problem behaviors for which unconditioned punishers were often applied. (p. 348)

This assertion is patently false as none of the studies cited directly compared the effects of these procedures to CESS and none of the problem behaviors approach severe or treatment refractory (Kayser et al., 2022). To suggest that the dimensions of behavior treated in a non-CESS study are equivalent to those treated in CESS studies is a logical fallacy. Proliferation of this notion by academic faculty should be considered a threat to the integrity of applied behavior analysis.

### Position Statements

Prior to the Report, the European Association for Behavior Analysis released a statement with no supporting evidence. The Association for Professional Behavior Analysts, Arizona Association for Behavior Analysis, California Association for Behavior Analysis, Massachusetts Association for Applied Behavior Analysis, and Oregon Association for Behavior Analysis released position statements containing false information (see Blenkush & O’Neill, 2022). In particular, Zarcone et al. (2020) was cited in support of statements against the use of CESS. The Report astutely identified errors made by Zarcone et al. in relation to side effects and experimental design but there has been no attempt to correct the article or position statements. Given the scientific foundation of applied behavior analysis, this should be of great concern to any member of those associations.

The ABAI Executive Council’s unprecedented decision to offer two position statements (Position A: opposed to CESS except under extreme circumstances; Position B: opposed under any condition) reflects the fact that no single position could be endorsed unanimously by all members of the Council. This warrants discussion as both positions should consist of logical extensions of report recommendations and should be consistent with existing position statements. In particular, the following excerpt from *The Right to Effective Behavioral Treatment*:

An individual has a right to the most effective treatment procedures available… A procedure's overall level of restrictiveness is a combined function of its absolute level of restrictiveness, the amount of time required to produce a clinically acceptable outcome, and the consequences associated with delayed intervention. (Van Houten et al., 1988)

Unfortunately, Position B was not a reflection of the Report recommendations nor consistent with the right to effective behavioral treatment. Instead, it appears to reflect an ideology and an attempt to placate a common criticism of applied behavior analysis. The latter is evidenced by repeated vague references to concerns raised by professional organizations and individuals. Furthermore, the finalized position statement notes that “both statements were *informed* by the report…”. However, the report concluded that CESS should only be implemented under highly restrictive conditions and that the evidence does not support a wholesale ban. We argue that Position A was *supported* by the report and Position B appears only to have been *informed* by the report. As it stands, the ABAI Position Statement on the use of CESS is a non sequitur.

## Limitations to the Standard of Care

As the Report noted, “severe problem behavior” is difficult to define and there is no agreed-upon nosological criteria. However, some researchers have acknowledged this problem and directed effort toward those ends (Morgan & Cox, 2021).

We believe the evidence for treatment refractory behaviors is overwhelming (see Blenkush & O’Neill, 2022) and it is important for applied behavior analysis to acknowledge, define, and address treatment-refractory behavior. Not as an excuse to use punishment procedures but to identify the prevalence and associated risk factors to inform the development of effective treatment. Our proposed definition is as follows: *A behavior for which a clinically significant outcome has not been observed, despite appropriate functional behavior assessments, and behavioral interventions administered with sufficient procedural integrity*. Risk factors include but are not limited to the individual’s: (1) treatment history (e.g., out-patient vs. in-patient hospitalization, day vs. residential behavioral treatment, psychotropic medication trials, restraint, health related supports, out-of-state placement); (2) medical/biological factors (e.g., rare congenital disorders; brain injury, psychosis); (3) physical attributes (e.g., size, strength, speed, and flexibility); (4) existing and potential for harm to self and/or others (e.g., risk of infection, maiming, and death); (5) regulatory restrictions (e.g., state-by-state prohibitions); and (6) limitations associated with the standard of care in applied behavior analysis.

To illustrate the need for such a definition, consider a study by Greer et al. (2016) cited as evidence that CESS is unnecessary (see Association for Professional Behavior Analysts [APBA], 2022). This study describes functional communication training applied across 20 individuals and demonstrates efficacy without punishment in all but one case. However, the participants were outpatients with a mean age of 7.7 years (2nd graders). We do not categorize such clients as severe or treatment refractory. A recent client started on CESS was in his 20s, well over 6-ft tall, weighed over 250 lb, and was unresponsive to over a decade of behavioral/psychopharmacological interventions. Emergency restraints requiring six to eight staff members were a daily part of his life.

### Functional Analysis

The Task Force outlined the necessity of functional analysis before introduction of standard treatments in behavior analysis. Functional analyses (for review, see Beavers et al., 2013) can often identify the maintaining variable(s) of problem behavior and inform the design of function-based treatments under highly controlled stimulus arrangements with specific treatment populations. However, there is a risk of false positives and ambiguous results (Rooker et al., 2014; Virues-Ortego et al., 2022). A substantial percentage of participants engage in automatic, unidentified, or multiply maintained problem behaviors (Iwata et al., 1994; Virues-Ortego et al., 2022). Example topographies at JRC include pulling out throat tissue, biting off a finger, biting off the tissue around the mouth and cheeks, auto-extraction of teeth, and rectal prolapsing.

Function identification does not, by default, indicate that clinically significant results are attainable, generalizable, or maintainable outside of the experimental setting (Foxx, 2016). Knowledge of a clear behavioral function does not necessarily ensure control of the relevant variables in the natural setting. Programmed reinforcers may fail to compete with the immediacy and magnitude of naturally occurring reinforcers such as pain attenuation or sensory stimulation (Virues-Ortego et al., 2022). The functional analysis literature on automatically maintained problem behaviors does little to quell these concerns:

Moreover, no study in the current review included a subsequent treatment analysis that was explicitly informed by patterns of responding during all FA conditions and that compared all potential treatments. Nevertheless, a perusal of studies that included treatment shows that, *in some cases* [emphasis added], hypotheses based on the results of the FA were confirmed (Virues-Ortego et al., 2022, p. 497).

Functional analysis is a process and is not limited in scope to the contrived conditions of a multi-element experimental design, although it is the most commonly cited approach (Jessel, et al., 2020). We conduct a variety of functional behavior assessments, in-depth direct observation and data collection in the natural setting, and have access to live and recorded video-monitoring across all contexts.

### Alternatives to CESS

**Fig. 1:**
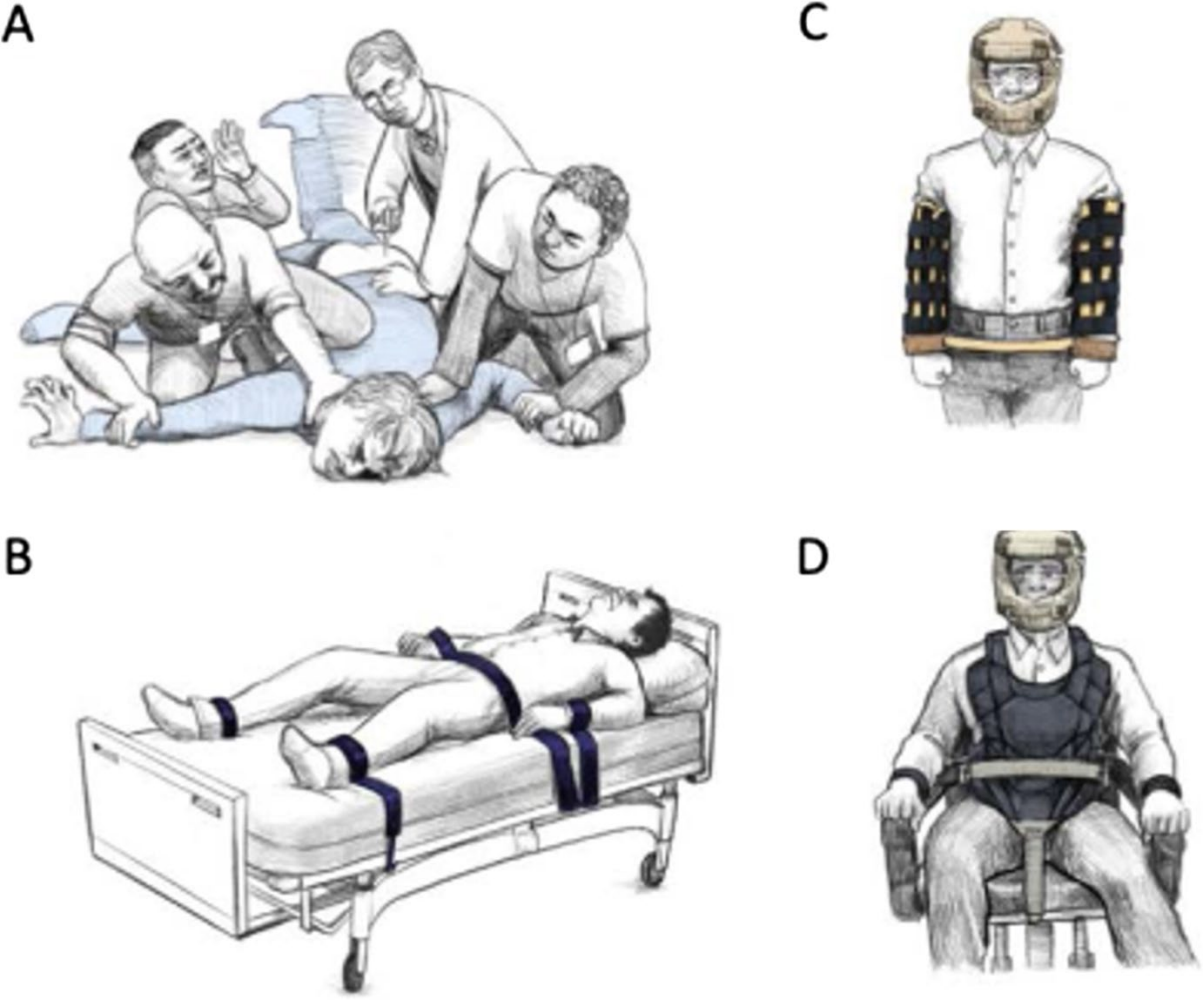
Illustration of **A**) physical restraint for an intramuscular injection of a psychotropic medication, **B**) mechanical bed restraint, **C**) helmet, arm splints, and waist restraint, and **D**) helmet, 5-point harness with wrist restraint

The Report outlined alternative treatments to CESS with a focus on differential reinforcement, skill-based instruction, extinction, antecedent-based interventions, psychotropic medications, and punishment procedures. However, individuals with severe problem behavior may also be exposed to other non-behavior analytic treatments, such as electroconvulsive therapy (Watchtel et al., 2011), deep-brain stimulation (Yan et al., 2022) psychosurgery (Anadan et al., 2004), and intramuscular injection of antipsychotic drugs. The Report included the use of protective equipment to maintain safety. However, physical and mechanical restraint may also be utilized (Blenkush & Cunningham, 2023). Figure 1 shows commonplace alternatives to CESS.

The promotion of absolute position statements in opposition to CESS moves treatment refractory clients towards a lifetime of psychopharmacology and restraint. These treatments have more risks and fewer benefits for treatment refractory behaviors when compared to CESS (Blenkush, 2017). There are innumerable alternative treatments for severe behaviors. However, we suggest there are no comparable *effective* alternatives for some clients with treatment refractory behaviors (Blenkush & O’Neill, 2020; O’Neill & Blenkush, 2020).

## Conclusion

Mulick and Butter (2016) noted “If a set of values or cultural biases prevents the objective assessment of variables of possible interest, science is short circuited; science is no longer practiced” (p. 304). We fear the ABAI Position Statement on CESS suppresses scientific inquiry; communicates false information; and ultimately harms clients with treatment-refractory behaviors by denying the very existence of their clinical problem and condemning a treatment option known to be efficacious. We suggest that questions about risks, benefits, and ethics are best addressed through individualized assessment, treatment, and the scientific process,not popular vote.

The interviews conducted with long-term residential care facilities found that they limit the number of “high-profile,” “intensive,” and “severe cases” but most providers reported they did not discharge clients due to the severity of their behavior. However, JRC has accepted and successfully treated, often without CESS or psychotropic medication, the severe problem behavior of individuals referred by many of the most highly regarded behavior analytic treatment facilities. We continue to do so. This is not simply a matter of facility capacities but one of treatment failure. The fact remains that we do not know how many individuals are rejected or expelled from behavioral treatment due to severe treatment-refractory problem behavior. We hold that there is a neglected population of individuals who are warehoused, deflected, or outright abandoned by applied behavior analysis. We should be willing to admit our limitations and acknowledge the uncomfortable truth that the evidence supports the use of CESS under certain rare conditions.
